# Human immature Langerhans cells restrict CXCR4-using HIV-1 transmission

**DOI:** 10.1186/1742-4690-11-52

**Published:** 2014-07-02

**Authors:** Ramin Sarrami-Forooshani, Annelies W Mesman, Nienke H van Teijlingen, Joris K Sprokholt, Michiel van der Vlist, Carla MS Ribeiro, Teunis BH Geijtenbeek

**Affiliations:** 1 Department of Experimental Immunology, Academic Medical Center, University of Amsterdam, Meibergdreef 9, 1105 AZ Amsterdam, The Netherlands; 2 Immune Regulation, Laboratory of Translational Immunology, University Medical Center Utrecht, Postbus 85500, 3508 GA Utrecht, The Netherlands

**Keywords:** HIV-1, Tropism, Coreceptor usage, CXCR4-using HIV-1 (X4), CCR5-using HIV-1 (R5), Selection of R5, Langerhans cells, *ex vivo* model, Infection, Transmission

## Abstract

**Background:**

Sexual transmission is the main route of HIV-1 infection and the CCR5-using (R5) HIV-1 is predominantly transmitted, even though CXCR4-using (X4) HIV-1 is often abundant in chronic HIV-1 patients. The mechanisms underlying this tropism selection are unclear. Mucosal Langerhans cells (LCs) are the first immune cells to encounter HIV-1 and here we investigated the role of LCs in selection of R5 HIV-1 using an *ex vivo* epidermal and vaginal transmission models.

**Results:**

Immature LCs were productively infected by X4 as well as R5 HIV-1. However, only R5 but not X4 viruses were selectively transmitted by immature LCs to T cells. Transmission of HIV-1 was depended on *de novo* production of HIV-1 in LCs, since it could be inhibited by CCR5 fusion inhibitors as well as reverse transcription inhibitors. Notably, the activation state of LCs affected the restriction in X4 HIV-1 transmission; immune activation by TNF facilitated transmission of X4 as well as R5 HIV-1.

**Conclusions:**

These data suggest that LCs play a crucial role in R5 selection and that immature LCs effectively restrict X4 at the level of transmission.

## Background

Human immunodeficiency virus-1 (HIV-1) is the virus causing acquired immunodeficiency syndrome (AIDS), which is a worldwide pandemic. With an estimated 34 million people infected worldwide, HIV-1 is a major health burden [1]. HIV-1 is a lentivirus that infects a variety of immune cells such as CD4^+^ T cells, macrophages and dendritic cells (DCs). Although CD4 is the main receptor for infection, HIV-1 also requires chemokine receptors for membrane fusion [2-4]. Chemokine receptor type 5 (CCR5) and C-X-C chemokine receptor type 4 (CXCR4) are the most important co-receptors for the two main HIV-1 variants, R5 and X4 viruses, respectively. HIV-1 infection predominantly occurs with the R5 HIV-1 strains. In contrast, X4 HIV-1 strains are rarely found during primary infection [5-9] even though X4 HIV-1 is present in chronic infected patients. During chronic infection, the virus tropism can switch from R5 to R5X4 or X4 viruses, which occurs in about 50% of infected individuals [10]. Switching of co-receptor usage is associated with an accelerated rate of loss of CD4 T cells resulting in rapid progression to AIDS and death [5-9]. Despite the presence of X4 viruses in the late stage of infection, X4 variants are rarely transmitted [5,8]. Indeed, both R5 and X4 HIV-1 variants have been detected in body fluids including semen, blood, and cervicovaginal secretions however only R5 HIV-1 variants are generally transmitted and establish the primary infection [11,12]. R5 HIV-1 selective transmission can indicate the existence of a “gatekeeper” that prevents transmission of X4 HIV-1 variants and/or a facilitator that supports transmission of R5 viruses [13,14], however, the underlying mechanisms remain unclear [15].

HIV-1 infection is categorized as a sexually transmitted disease as more than 85% of HIV-1 infection occurs via sexual contacts [16,17]. For transmission, HIV-1 needs to cross female and male genital, and intestinal mucosal epithelium [18-21]. Langerhans cells (LCs) are a subset of DCs that line the mucosa the genital tracts and are therefore the first immune cells to encounter HIV-1 [22,23]. There are several reports that highlight a role for LCs in HIV-1 transmission [23-26]. LCs act as a natural barrier against HIV-1 that capture HIV-1 through the C-type lectin langerin leading to internalization and degradation into Birbeck granules, limiting infection [27]. However blockage, saturation of langerin or inflammatory conditions lead to the infection of LCs and these infected LCs efficiently transmit HIV-1 to T cells [27-33].

LCs express HIV-1 receptor CD4 and the co-receptor CCR5 [34,35]. Therefore it is expected that LCs are mainly infected by R5 HIV-1 strains [24]. Several studies have shown that LCs, under steady state, can only be infected with R5 HIV-1 and transmit R5 viruses [24,36] but not X4 viruses [32,37].

Here we have investigated whether primary LCs play a role in the selective transmission of R5 HIV-1 variants and the mechanism underlying this selection. We have used an *ex vivo* tissue transmission model to investigate transmission of X4 and R5 HIV-1 by LCs. Notably, our data show that both variants infect LCs but immature LCs selectively transmit R5 HIV-1 to target cells. Immune activation changed this restriction and allowed transmission of both X4 and R5 viruses by LCs. Thus, immature LCs have an intrinsic restriction mechanism preventing transmission of X4 HIV-1, which is abrogated upon immune activation. Identification of this restriction mechanism in LCs might provide novel targets for preventing sexual HIV-1 transmission.

## Results

### Human primary LCs transmit predominantly R5 HIV-1

**Figure 1:**
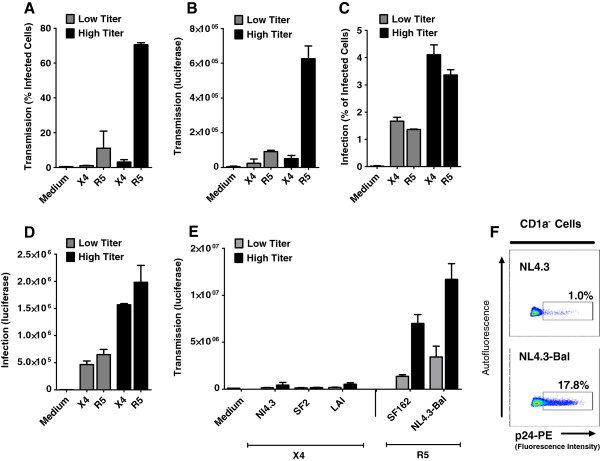
**Human primary LCs predominantly transmit R5 HIV-1. (A-B)** Human epidermal sheets were pulsed with low (4000 IU or 40 ng HIV-1 p24) or high (20000 IU or 400 ng HIV-1 p24) titers of HIV-1 NL4.3 (X4) or HIV-1 NL4.3-Bal (R5) for 5 hours **(A)** At day 3, emigrated LCs were cocultured with CCR5 vector-transduced Jurkat (CCR5 Jurkat T cells) and infection of Jurkat cells was measured at day 6 by intracellular p24 staining or GFP expression. T cell-marker CD3 and LC-marker CD1a were used to exclude LCs and LCs-T cells conjugates from analysis. Error bars represent the mean ± SEM of at least 3 independent experiments. **(B)** Emigrated LCs were cocultured with TZM-bl cells for 2 days and infection was determined by measuring luciferase activity (relative luciferase units [RLU]). Error bars represent the mean ± SEM of at least 3 independent experiments. **(C-D)** CCR5 Jurkat T cells (C) and TZM-bl cells (D) were infected with low or high titers of X4 and R5 virus, and infection was determined by intracellular p24 staining or luciferase activity respectively at day 2. Error bars represent the mean ± SEM of triplicates. **(E)** Epidermal sheets were infected with different X4 (HIV-1 SF2, LAI and NL4.3) and R5 viruses (SF162 and HIV-1 NL4.3-Bal). Emigrated LCs were cocultured with TZM-bl cells and infection of TZM-bl cells was determined by luciferase activity. Error bars represent the mean ± standard errors of the mean (SEM) of at least 3 independent experiments. **(F)** Vaginal LCs were exposed to X4 and R5 HIV-1 and after 3 days were co-cultured with CCR5 Jurkat T cells. Transmission was determined by measuring infection of CCR5 Jurkat T cells by intracellular p24 staining after 3 days. Dotplots represents two independent experiments/donors.

We have used an *ex vivo* tissue transmission model [30] to investigate the role of LCs in transmission of X4 and R5 HIV-1. Human epidermal sheets were exposed to different titers of X4 and R5 HIV-1, NL4.3 and NL4.3-BaL, respectively. After 5 hours, unbound virus was washed away and infected sheets were cultured for 3 days. Migrated LCs were harvested and cocultured with CCR5 vector-transduced Jurkat T cells (CCR5 Jurkat T cells) that are permissive for both R5 and X4 HIV-1. After 3 days transmission was determined by measuring infection of CCR5 Jurkat T cells by intracellular p24 staining. R5 HIV-1 was efficiently transmitted by LCs to CCR5 Jurkat T cells (Figure 1A). In contrast, transmission of X4 HIV-1 by LCs was low, even when high titers were used. To exclude that target cell characteristics affected HIV-1 transmission, we also investigated HIV-1 transmission from LCs to another target cell-line TZM-bl, which is also susceptible to both R5 and X4 HIV-1 [38]. Similarly, LCs transmitted R5 viruses to TZM-bl cells much more efficiently than X4 HIV-1 (Figure 1B). The predominant R5 HIV-1 transmission was not due to selective infection of the target cells, since both CCR5 Jurkat T cells and TZM-bl cells were efficiently infected by X4 and R5 HIV-1 (Figure 1C and D). To confirm that the predominant transmission of R5 HIV-1 by LCs was not dependent on the HIV-1 strains, epidermal sheets were infected with additional X4 (SF2, LAI) and R5 (SF162) strains. Similarly to NL4.3 and NL4.3-BaL, HIV-1 SF162 was transmitted more efficiently than HIV-1 SF2 and LAI strains (Figure 1E). Next, we isolated LCs from vaginal mucosa and investigated transmission by these LCs. Similar to epidermal LCs, vaginal LCs selectively transmitted R5 HIV-1 (Figure 1F). These data strongly suggest that primary human LCs efficiently transmit R5 but not X4 HIV-1 variants to T cells.

### LCs are infected by X4 HIV-1

**Figure 2:**
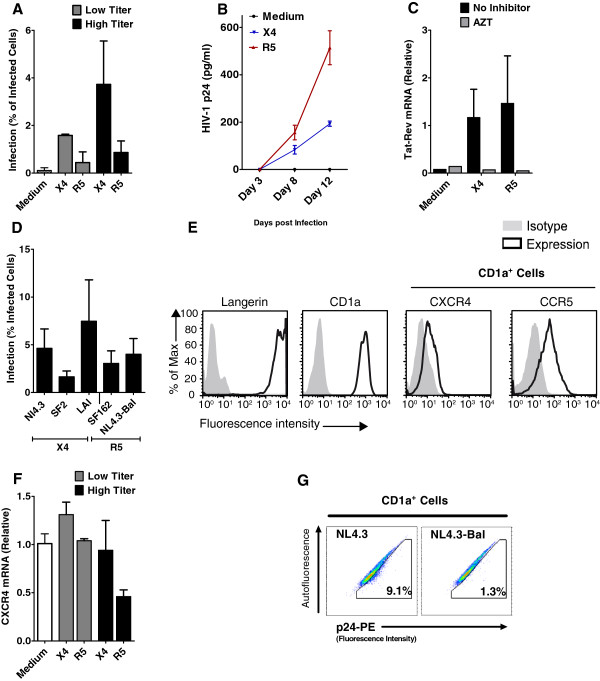
**Human primary LCs are infected by X4 HIV-1 variants. (A-B)** Human epidermal sheets were pulsed with low or high titers of HIV-1 NL4.3 (X4) or HIV-1 NL4.3-Bal (R5) for 5 hours, washed and cultured for 3 days. **(A)** Infection of emigrant LCs was determined by intracellular p24 staining or GFP expression in combination with LC-marker CD1a by flow cytometric analysis. The percentage of CD1a^+^p24^+^ cells are depicted here as % of infected cells. Error bars represent the mean ± SEM of at least 3 independent experiments. **(B)** Supernatant of cultured emigrant LCs was collected at day 3, 8 and 12 post-infection and HIV-1 p24 was measured in the supernatant by ELISA. Error bars represent the mean ± SEM of duplicates. **(C)** Epidermal sheets were pulsed with HIV-1 NL4.3 or HIV-1 NL4.3-Bal in the presence or absence of AZT for 5 hours. At day 3, HIV-1 tat/rev transcription in emigrated LCs was analyzed by real-time qPCR. Error bars represent the mean ± SEM of duplicates. One experiment representative of three is presented. **(D)** Epidermal sheets were pulsed with different X4 (HIV-1 SF2, LAI and NL4.3) and R5 viruses (SF162 and HIV-1 NL4.3-Bal) and after 3 days infection of LCs was determined. Error bars represent the mean ± SEM of at least 3 independent experiments. **(E)** Surface expression of HIV-1 coreceptors CCR5 and CXCR4 on immature LCs. Histograms represent at least 3 donors. **(F)** Epidermal sheets were pulsed with X4 and R5 for 5 hours. After 3 days mRNA expression of CXCR4 was measured in emigrant LCs by real-time qPCR. Error bars represent the mean ± SEM of duplicates. **(G)** Vaginal LCs were infected with HIV-1 NL4.3 and NL4.3-Bal and infection was measured by intracellular p24 staining. Representative dotplots of one out of two donors are shown.

Next we investigated infection of LCs by X4 and R5 HIV-1 variants. Epidermal sheets were infected with X4 and R5 HIV-1 for 5 hours and epidermal sheet were washed extensively and cultured for 3 days. At day 3, migrated LCs were harvested and cultured for 3 additional days. LC infection was analyzed by intracellular p24 expression in combination with LC-marker CD1a and T cell-marker CD3. The majority of cells that migrated were LCs. Hardly any T cells were present and these T cells were not infected by HIV-1 [30]. Notably, LCs were infected by both X4 and R5 HIV-1 variants. In fact, infection with X4 HIV-1 was more efficient (Figure 2A). Similar results were obtained with NL4.3-eGFP (X4) and NL4.3-eGFP-BaL (R5) that express GFP only upon replication, further supporting viral replication of both X4 and R5 HIV-1 variants in LCs (data not shown).Next we investigated whether LCs were productively infected by measuring HIV-1 p24 in the supernatant. Epidermal sheets were exposed to X4 and R5 HIV-1 variants. After 3 days, emigrated LCs were collected and cultured for several days, and p24 HIV-1 was measured in the supernatant by ELISA. Both X4 and R5 HIV-1 variants show productive infection as observed by p24 production (Figure 2B). In addition, HIV-1 tat/rev transcription was analyzed by quantitative real-time PCR. HIV-1 tat/rev transcription of X4 HIV-1 in LCs was similar to that of R5 HIV-1 (Figure 2C). To demonstrate that replication was required, epidermal sheets were treated with HIV-1 reverse transcriptase inhibitor AZT prior to infection. No tat/rev was detected for the cells that were treated with AZT (Figure 2C). In order to control for possible differences in infection of selected viruses, epidermal sheets were also infected with HIV-1 LAI, SF2 and SF162 strains. Exposure of epidermal sheets with HIV-1 LAI and SF2 also revealed infection of LCs with X4 HIV-1 strains in a level comparable to R5 HIV-1 strains (Figure 2D). In accordance with the infection data, immature LCs express both CCR5 and CXCR4 as measured by flow cytometry (Figure 2E). Furthermore expression of CXCR4 mRNA was detected in emigrated LCs from uninfected and infected epidermal sheets (Figure 2F). Similar to epidermal LCs, vaginal LCs were infected by both X4 and R5 HIV-1 (Figure 2G). Thus, these data strongly suggest that primary LCs are infected by both X4 and R5 HIV-1 and that the predominant transmission of R5 HIV-1 by LCs is not due to inability of X4 HIV-1 to infect LCs.

### Activated LCs efficiently transmit X4 HIV-1 variants

**Figure 3:**
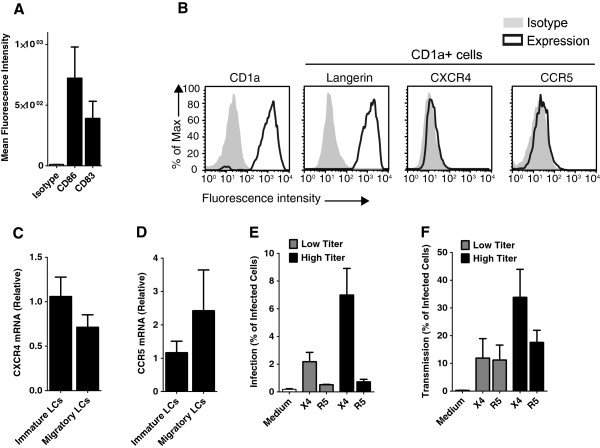
**Activated LCs efficiently transmit X4 HIV-1 variants. (A)** Migratory LCs were analysed for expression of CD83 and CD86 by flow cytometry. Error bars represent the mean ± SEM of at least 3 different donors. **(B)** Surface expression of HIV-1 coreceptors on migratory LCs was determined by CCR5 and CXCR4 staining in combination of LC-markers CD1a and langerin by flow cytometry. **(C-D)** Immature LCs and migratory LCs were isolated and harvested and mRNA expression of CCR5 **(C)** and CXCR4 **(D)** was measured by real-time qPCR. Error bars represent the mean ± SEM of at least 3 donors. **(E)** Migratory LCs were infected with different titers of NL4.3 (X4) and NL4.3-Bal (R5) HIV-1. After 3 days infection of LCs was determined by intracellular HIV-1 p24 staining or GFP expression in combination with LC-marker CD1a by flow cytometric analysis. Error bars represent the mean ± SEM of at least 3 independent experiments. **(F)** Migratory LCs were pulsed with X4 or R5 HIV-1 strains and after 3 days, cocultured with CCR5 Jurkat T cells for additional 3 days. LCs mediated HIV-1 transmission was determined via measuring infection of CCR5 Jurkat T cells by intracellular p24 staining or GFP expression in combination with T cell-marker CD3 and LC-marker CD1a following flow cytometry. Error bars represent the mean ± SEM of at least 3 independent experiments.

Next we investigated whether activation of cells affects HIV-1 selection during transmission by LCs. Epidermal sheets were cultured for 3 days and the migratory LCs were harvested. These migratory LCs have an activated phenotype as shown by increased expression of CD83 and CD86 (Figure 3A). The migratory LCs also expressed CCR5 and CXCR4 (Figure 3B) although at a lower level than immature LCs (Figure 2E). The mRNA levels of both CCR5 and CXCR4 in migratory LCs were similar to those observed in immature LCs (Figure 3C and 3D). Next migratory LCs were infected with both HIV-1 strains and infection was measured by flow cytometry. Similar to the *ex vivo* infected LCs (Figure 2A), migratory LCs were infected by both X4 and R5 HIV-1 variants and infection with X4 HIV-1 was higher than R5 HIV-1 (Figure 3E). These data suggest that the expression of the co-receptors is not restricting infection of migratory LCs even though the expression of the co-receptors is lower. Next we investigated transmission of HIV-1 by these activated LCs. Migratory LCs were infected with X4 and R5 HIV-1, washed and cultured for 3 days. At day 3, target cells were added and infection of the target cells was analyzed by p24 intracellular staining. Notably, in contrast to the *ex vivo* model, migratory LCs transmitted both X4 and R5 HIV-1 variants (Figure 3F). These data suggest that activation of LCs allows transmission of X4 HIV-1 to T cells.

### Virus replication is necessary for HIV transmission

**Figure 4:**
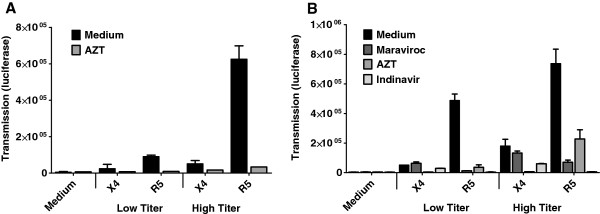
**Virus replication is necessary for HIV transmission. (A)** Epidermal sheets were pulsed with low or high titers of HIV-1 NL4.3 (X4) or HIV-1 NL4.3-Bal (R5) in the presence or absence of the reverse transcriptase inhibitor AZT. After 3 days, emigrated LCs were cocultured with TZM-bl cells for 2 days. HIV-1 transmission to TZM-bl cells was determined by measuring luciferase activity (luminescence value or relative luciferase units [RLU]). Error bars represent the mean ± SEM of triplicates. **(B)** Migratory LCs were infected with X4 or R5 HIV-1 strains in presence or absence of different inhibitors including CCR5 antagonist Maraviroc, AZT and protease inhibitor Indinavir. After 3 days culture, LCs were cocultured with TZM-bl cells. After 2 days, transmission was measured by luciferase assay. Error bars represent the mean ± SEM of triplicates.

Next we investigated whether transmission by immature and migratory LCs was dependent on replication. Epidermal sheets were infected with HIV-1 in presence or absence of the reverse transcriptase inhibitor AZT. Emigrated LCs were cocultured with TZM-bl cells and transmission was measured. AZT completely prevented transmission of HIV-1 by LCs (Figure 4A). These data strongly suggest that HIV-1 replication in immature LCs is required for HIV-1 transmission. Next, migratory LCs were infected with HIV-1 strains in presence or absence of different inhibitors, including CCR5 antagonist Maraviroc, AZT and protease inhibitor Indinavir, which prevents HIV-1 release. Both AZT and Indinavir blocked transmission of both R5 and X4 HIV-1 whereas Maraviroc only blocked transmission of R5 HIV-1 (Figure 4B). These data indicate that HIV-1 transmission by both immature and mature LCs is dependent on virus replication.

### TNF-matured LCs transmit both X4 and R5 HIV-1

**Figure 5:**
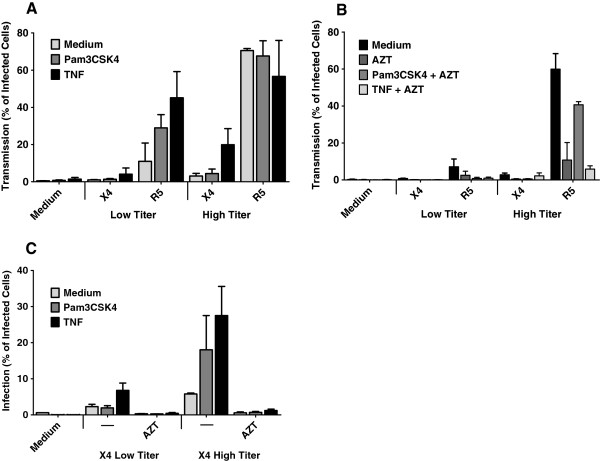
**TNF-matured LCs transmit both X4 and R5 HIV-1. (A)** Epidermal sheets were pretreated with different stimuli including Pam3CSK4 and TNF for 4 hours, pulsed with low and high titers of X4 and R5 HIV-1 for 5 hours **(A)** At day 3 emigrant LCs were collected, cocultured with CCR5 Jurkat T cells and HIV-1 transmission was determined after 3 days by measuring intracellular HIV-1 p24 or GFP expression by flow cytometry. Using T cell-marker CD3 and LC-marker CD1a, infection of the target cells were exclusively analyzed. Error bars represent the mean ± SEM of at least 3 independent experiments. **(B)** Epidermal sheets were pretreated with Pam3CSK4 and TNF for 4 hours or left untreated as control, following exposure to low or high titers of X4 and R5 HIV-1 in presence or absence of AZT for 5 hours. After 3 days, emigrated LCs were cocultured with CCR5 Jurkat T cells and transmission rate was determined at day 6 by flow cytometry. Error bars represent the mean ± SEM of at least 3 independent experiments. **(C)** Treatment of LCs with stimuli highly increased infection. Epidermal sheets were pretreated with Pam3CSK4 and TNF for 4 hours then pulsed with X4 HIV-1 for 5 hours. Infection of emigrant LCs was determined at 6–7 days by intracellular p24 staining in combination with LC-marker CD1a by flow cytometric analysis. Error bars represent the mean ± SEM of 3 independent experiments.

Compared to immature LCs, migratory LCs are activated and express high levels of the maturation markers CD86 and CD83 [30]. Since the activation state is the main difference between *ex vivo* explants and migratory LCs transmission models, we studied the effect of pre-activation of LCs with TLR-2 agonist, Pam3CSK4 [39] and TNF on X4 HIV-1 transmission. Epidermal sheets were pretreated with different stimuli including Pam3CSK4 and TNF and infected with X4 and R5 HIV-1. After 3 days, migrated LCs were harvested and cocultured with target cells to investigate transmission. Notably, TNF induced transmission of X4 HIV-1 by LCs (Figure 5A) and this was dependent on viral replication in LCs since AZT abrogated transmission (Figure 5B). Pam3CSK4 similarly to TNF increased the transmission rate of R5 HIV-1. AZT did not block HIV-1 transmission by Pam3CSK4 stimulated LCs when high titer of the R5 virus were used. These data are in concordance with our previous study that Pam3CSK4 enhances the capture by LCs, and therefore increases HIV-1 transmission independent of HIV-1 infection [30]. Although Pam3CSK4 similarly to TNF increased the transmission rate of R5 HIV-1 variants, its effect on X4 HIV-1 variants was not noticeable. Previously we have shown that TNF enhances infection of LCs by R5 HIV-1, and thereby increases transmission of R5 HIV-1 [30]. Our results show that TNF also increased the infection rate of LCs with X4 HIV-1 (Figure 5C) and infection was blocked by AZT. These data strongly suggest that immune activation of LCs is able to abrogate the restriction of X4 HIV-1 transmission.

## Discussion

CCR5-using HIV-1 is the predominant strain being transmitted, suggesting that part of the R5 selection occurs at the mucosa of vaginal tissues. Here we have shown that primary LCs express CXCR4 and CCR5, and become infected by both X4 and R5 HIV-1. However, only R5 HIV-1 is transmitted by primary LCs using an *ex vivo* tissue transmission model. These data strongly suggest there is restriction in the transmission of X4 HIV-1 by LCs. Immune activation abrogates this restriction since activated LCs transmit both X4 and R5 HIV-1.

In general R5 viruses are associated with HIV-1 transmission and predominate during the early stages of infection [40,41]. During disease progression, X4 HIV-1 populations have been detected in about 50% of the patients. Recent studies show that indeed so-called founder/transmitted viruses are R5 and in some cases dual X4R5 but not X4 variants [13,14]. These studies suggest that there are likely several mechanisms for R5 selection [15]. Heterosexual transmission is the main route of infection, suggesting that HIV-1 after sexual contact needs to pass the mucosal vaginal barrier to infect target cells. Little is known about the selectivity during transmission over the mucosal barrier but it is assumed that both X4 and R5 viruses are challenged by this mechanical barrier [15]. LCs are present in the epithelial layer of mucosa and are as antigen presenting cells ideally positioned as well as equipped to capture incoming pathogens [42]. Immature LCs are not permissive to infection and have been shown to present another barrier for HIV-1 through the function of the C-type lectin langerin, which captures both X4 and R5 viruses, leading to HIV-1 internalization and degradation [27,30]. Immune activation or high virus titers allow infection of LCs with R5 [27,30] and transmission of R5 HIV to T cells. The major route of transmission by LCs requires productive infection of LCs and production of virus particles, known as *cis* infection [27,36]. We observed that transmission by LCs is dependent on productive infection, in line with previous findings. Immune activation has been shown to allow *trans* infection, which is replication independent and relies on capture and transmission to other cells [30,32]. Consistent with previous publications [24,25,27-30,33,43], our results confirmed that LCs selectively transmit R5 HIV-1 when they are exposed to virus *ex vivo*. In general, it is thought that LCs express HIV-1 receptor CD4 and CCR5 coreceptor, which allows productive infection with only R5 HIV-1 and selective transmission of R5 strains through a *cis* pathway [24,32,34,36,44,45]. Several studies have used different models for LCs such as cell-lines or *in vitro* generated monocyte-derived LCs [32,46] and these might have distinct chemokine receptor expression than primary LCs. We have used the *ex vivo* tissue transmission model [30] and observed that emigrated LC were infected by different X4 viruses. Some reports are in accordance with our study and have shown that LCs become infected by X4 HIV-1 [47,48]. However, these studies did not observe a restriction in X4 transmission by LCs. The discrepancy between our study could be differences in LC activation state or source. Tchou et al. [48] stimulated epidermal LCs with GM-CSF prior to infection which might activate LCs, whereas Sivard et al. [47] used CD34^+^ progenitor-derived LCs.

Immature LCs express the CXCR4 coreceptor as has also been shown by others [48,49]. These data suggest that permissiveness to infection for R5 viruses might not be the underlying mechanism for R5 selection. In fact, R5 selection might occur at the transmission phase. Infection of LCs with both X4 and R5 was dependent on replication and could be inhibited by fusion or RT inhibitors. Similarly, transmission of R5 was dependent on replication, suggesting that infection *in trans* did not account for the selection. Indeed, C-type lectin receptors such as DC-SIGN and langerin are not selective in their binding of X4 and R5 viruses [50-52] further suggesting that selection is not due to differences in binding. We observed that LCs were more efficiently infected by NL4.3 (X4) compared to NL4.3-BaL (R5), suggesting that the level of infection did not affect transmission.

Migratory LCs, which have an activated phenotype, were infected by both X4 and R5 and notably were able to transmit X4 as well as R5. Migratory LCs showed lower expression of co-receptors compared to immature LCs. However the lower co-receptor expression did not affect infection of LCs with both X4- and R5-using viruses. Efficient transmission of X4 HIV-1 by infected migratory LCs suggests that immune activation and subsequent infection might allow X4 transmission. Interestingly LCs in *ex vivo* model after treatment with TNF were able to transmit X4 HIV-1 variants. Although TNF enhances infection of LCs [30,53], our data suggest that the level of infection does not affect transmission, since X4 viruses efficiently infected LCs butt were not transmitted to T cells. It is possible that immune activation changes the viral internalization pathway or vesicle transport in LCs, allowing efficient transmission. Of note is that infection needs to occur at the mature/activated state to observe X4 HIV-1 transmission, since we did not observe X4 transmission by mature LCs that had been infected *ex vivo* in an immature state. Epidermal sheets that were pretreated with Pam3CSK4, even after treatment with AZT, were able to transmit HIV-1 R5 HIV-1 variants. This effect of Pam3CSK4 was expected as it was previously shown that Pam3CSK4 increases *trans* infection by LCs [30]. However HIV-1 X4 variants were not transmitted even through *trans* pathway. These data suggest that not only *cis* infection but also *trans* infection of X4 viruses is inhibited by immature LCs. These data strongly suggest that R5 selection by immature LCs is dependent on X4 restriction that prevents transmission but not infection of LCs. There is a recent report suggesting that mature DCs produce SDF-1/CXCL12, which inhibits the propagation of X4 HIV-1 isolates at the DC-T-cell infectious synapse [54]. We have investigated the expression of CXCL12 and mature LCs expressed higher levels of CXCL12 than immature LCs (data not shown). Moreover, we did not observe any inhibition of the supernatant from immature and mature LCs on infection of target cells with X4-using viruses (data not shown). These data strongly suggest that the restriction is not a soluble factor but a mechanism intrinsic to immature LCs. Future investigations are required to figure out the mechanism underlying this selection.

## Conclusions

In summary, this study show that HIV-1 CXCR4-using variants are able to infect LCs. Although immature LCs selectively transmit HIV-1 CCR5-using strains, this selection is not due to permissiveness to infection. Identification of the X4 restriction mechanism by LCs might enable us to develop strategies to also prevent R5 transmission. Identification of HIV-1 X4 inhibitor(s) may lead to better understanding of HIV-1 transmission and more importantly a step forward for prevention and/or treatment of HIV-1 infection.

## Methods

### Antibodies and reagents

The following reagents were used: KC57-RD1-PE (anti–HIV-1 p24; Beckman Coulter), HI149-FITC (anti-CD1a; Pharmingen), HI149-APC (anti-CD1a; BD biosciences), NA1/34 (anti-CD1a; Dako Cytomation), UCHT1-PE (anti CD3; eBioscience), SP34-2- PercP (anti CD3; BD Pharmingen), 2D7-PE and 2D7-APC (anti-CCR5; Pharmingen), 12G5-PerCP and 12G5-PE (anti CXCR4; R & D System), SK3-FITC (anti-CD4; BD biosciences), RPA-T4 (anti-CD4; Biolegend), DCGM4-PE (anti CD207, Beckman Coulter), 12D6 (anti CD207; Novocastra), HB15a-PE (anti CD83; Beckman Coulter), 2331-FITC and 2331-PE (FUN-1) (anti CD86; BD Pharmingen), IgG PE isotype (BD Biosciences), tripalmitoylated lipopeptide Pam3CSK4 (Invivogen), recombinant human TNF (Strathmann Biotec). The following HIV-1 inhibitors were obtained through the NIH AIDS Reagent Program, Division of AIDS, NIAID, NIH: Maraviroc, Indinavir and Zidovudine (AZT).

### Plasmids and cell lines

pNL4.3eGFP and pNL4.3eGFP-BaL were generously provided by C. Aiken, Vanderbilt University, Nashville, Tennessee, USA. The human CCR5 lentiviral vector pLOX (LV-CCR5) was generously provided by V. Piguet, University Hospital and Medical School of Geneva, Geneva, Switzerland [55,56]. Jurkat T cells expressing CCR5 were generated by retroviral transduction as previously described [55,56].

### Viruses

293 T cells were transfected with NL4.3-BaL or NL4.3-eGFP-BaL proviral plasmids (10 μg). At day 2, viruses were harvested. The following viruses were obtained through the NIH AIDS Reagent Program, Division of AIDS, NIAID, NIH: HIV-1_LAI_ from Dr. Jean-Marie Bechet and Dr. Luc Montagnier [57,58]. HIV-1 SF_2_ and HIV-1 SF_162_ from Dr. Jay Levy [59,60]. Viruses stocks were propagated on PHA-stimulated human PBMCs. All produced viruses were quantified by p24 ELISA (Perkin Elmer Life Sciences) and titrated using the indicator cells TZM-bl (contributed by John C. Kappes, Xiaoyun Wu [both at University of Alabama, Birmingham, Alabama, USA], and Tranzyme Inc. through the NIH AIDS Research and Reference Reagent Program) [30].

### *Ex vivo* model

Human tissues were obtained from healthy donors undergoing corrective breast or abdominal surgery. The study was approved by Medical Ethics Review Committee in accordance with the ethical guidelines of the Academic Medical Center. Epidermal sheets were prepared as described previously [27]. Briefly, skins were cut 3-mm-thick slices, containing the epidermis and dermis, using a dermatome. The slices were incubated with Dispase II (1 mg/ml, Roche Diagnostics) in Iscoves Modified Dulbecco’sMedium (IMDM), 10% FCS and gentamycine (10 mg/ml) for either 1 h at 37 C or overnight at 4 C. Epidermis were mechanically separated, washed in IMDM medium and cut it into 1-cm^2^ pieces and were used for *ex vivo* experiments. LC-enriched epidermal single-cell suspensions were generated as described before [27]. Briefly, epidermal sheets were incubating in PBS containing DNase I (20 units/ml; Roche Applied Science) and trypsin (0.05% Beckton Dickinson) for 30 min at 37 C. Trypsin digestion was inactivated with FCS. Through repeated pipetting of the digested epidermal sheets and filtration through sterile mesh, a single-cell suspension was generated. Single-cell suspension was then layered on Ficoll gradient and immature LCs were purified using CD1a-labeled immunomagnetic microbeads (Miltenyi Biotec). Isolated LCs (99% CD1a^+^, langerin+) were tested for expression of HIV-1 related cell surface markers.

### Vaginal LC

Vaginal mucosa was obtained from routinely discarded tissue of vaginal prolapse surgeries. The study was approved by Medical Ethics Review Committee in accordance with the ethical guidelines of the Academic Medical Center. After incubation with Dispase II (3 mg/mL, Roche Diagnostics) in IMDM, vaginal mucosal sheets were separated from submucosa and further cultured in IMDM supplemented with 10% FCS, gentamycine (10 mg/mL), penicillin (2500 U/ml), streptomycin (2500 mg/ml), and L-Glutamine (100 mmol/l) until disintegration of the tissue. Further vaginal LC purification was performed using a Ficoll gradient and CD1a microbeads (Miltenyi Biotec).

### Stimulation

Epidermal sheets were incubated with Pam3CSK4 (5 μg/ml) or TNF (0.1 μg/ml) for 4 hours prior to infection. TNF was titrated in the *ex vivo* experiments for optimal HIV-1 transmission, Pam3CSK4 was titrated for optimal HIV-1 infection of CCR5 Jurkat cells and the other ligands were used at concentrations that activate DCs [61,62].

### Infection and transmission assay using the *ex vivo* model

For infection, human epidermal sheets were inoculated with low (4.0E + 03 IU or 4.0E + 01 ng HIV-1 p24) or high (2.0E + 04 IU or 4.0E + 02 ng HIV-1 p24) titers of different HIV-1 strains. After 5 hours incubation, infected sheets were extensively washed and cultured in fresh media for 3 days. For treatment with HIV-1 inhibitors, the sheets were pre-incubated with AZT (10 uM), Indinavir (1 uM) or Maraviroc (4 uM) for one hour before infection. The sheets remained with the HIV-1 inhibitors for 3 days (the day of transmission assay). At day 3, the epidermal sheets were removed and emigrated LCs were harvested. Emigrated LCs were cultured for several days for infection assays or were used for transmission assay. For transmission assay emigrated LCs were cocultured with either CCR5+ Jurkat T cells (5.0E + 04 cells) or TZM-bl cells (70-80% confluence in 96 wells) for 3 and 2 days, respectively. Following methods were used for monitoring HIV-1 infection in the emigrated LCs: Intracellular HIV-1 p24 staining or GFP expression in combination with LC-marker CD1a by flow cytometric analysis (6 days post infection), measurement of p24 in culture supernatants at different time points by ELISA (Perkin Elmer Life Sciences) and real-time qPCR for HIV-1 tat/rev transcription on the mRNA extracted from LCs lysates (3 days post infection). LCs-mediated transmission of HIV-1 to CCR5+ Jurkat cells were determined by intracellular p24 staining or GFP expression in combination with LC-marker CD1a and T cell-marker CD3 by flow cytometry after 3 days coculturing. Transmission to TZM-bl cells was evaluated by measuring luciferase activity in the cocultures at 2 days post transmission, using a luciferase reporter assay kit (Promega).

### Migratory LCs

Migratory LCs were generated by floating the epidermis on IMDM, 10% FCS, 10 mg/ml gentamycin. After 3 days migratory LCs were harvested, layered on Ficoll gradient and cultured at 5.0E + 05 cells/ml in IMDM, 10% FCS and 10 mg/ml gentamycine. For infection, 5.0E + 04 migratory LCs were exposed to low (4.0E + 03 IU, MOI 0.08) or high titer (2.0E + 04 IU, MOI 0.4) of different HIV-1 variants. For treatment with HIV-1 inhibitors, the migratory LCs were pre-incubated with AZT (10 uM), Indinavir (1uM) or Maraviroc (4 uM) for one hour before infection. The infected cells remained with the HIV-1 inhibitors for 3 days (day of transmission). After 3 days LCs were harvested and extensively washed. Migratory LCs were incubated for 3 additional days for determination of infection or were coculotured with the target cells similar to above described for *ex vivo* model.

### Real-time qPCR

LCs were extensively washed with PBS. Both host mRNA and viral RNA were specifically isolated with an mRNA Capture kit (Roche) and by an additional 1 h of incubation in streptavidin-coated plates (Sigma) to ensure complete removal of complexes of mRNA and biotin-labeled oligo(dT). cDNA was synthesized with a reverse-transcriptase kit (Promega). Samples were amplified by PCR with SYBR Green as described [63]. Specific primers for HIV-1Tat/ Rev, CXCR4 and GAPDH [63] were designed by Primer Express 2.0 (Applied Biosystems). The sequences are as follows: HIV-1 Tat-Rev, forward, ATGGCAGGAAGAAGCGGAG, reverse, ATTCCTTCGGGCCTGTCG; CXCR4 forward, CAACGTCAGTGAGGCAGATGA, CXCR4, reverse, TACCAGGCAGGATAAGGCCAA. Transcription was normalized to GAPDH transcription. For Tat-Rev, the relative viral expression of X4 HIV-1 infected sample were set at 1 whereas for CXCR4, mRNA expression of non infected samples was set at 1.

## Abbreviations

HIV-1: Human immunodeficiency virus type-1; LCs: Langerhans cells; DCs: Dendritic cells; MDDCs: Monocyte derived dendritic cells; X4: CXCR4-using HIV-1; R5: CCR5-using HIV-1; IU: Infection unit; FFU: Focus forming unit.

## Competing interests

The authors declare that they have no competing interests.

## Authors’ contributions

RSF designed aspects of this study, executed and interpreted most experiments and prepared the manuscript. AWM provided help with execution of some experiments and helped prepare the manuscript. NHvT set up the vaginal LC isolation and provided help for execution of experiments with vaginal LCs. JKS provided help with isolation of LC mRNA. MvdV provided help with designing experiments and interpretation of experiments. CMSR provided help with designing experiments, interpretation of experiments and helped prepare the manuscript. TBHG supervised all aspects of this study and helped prepare the manuscript. All authors read and approved the final manuscript.
